# Non-metabolic enzyme function of PKM2 in hepatocellular carcinoma: A review

**DOI:** 10.1097/MD.0000000000035571

**Published:** 2023-10-20

**Authors:** Shuangxia Zhang, Zhangxiu Liao, Shubo Li, Ying Luo

**Affiliations:** a School of Pharmacy, Youjiang Medical University for Nationalities, Baise, Guangxi, China; b Basic Medical College, Youjiang Medical University for Nationalities, Baise, Guangxi, China; c Key Laboratory of Right River Basin Characteristic Ethnic Medicine Research in Guangxi, Baise, Guangxi, China; d Key Laboratory of Tumor Immunopathology, Youjiang Medical University for Nationalities, Baise, Guangxi, China.

**Keywords:** hepatocellular carcinoma, non-metabolic enzyme function, pyruvate kinase M2

## Abstract

Hepatocellular carcinoma (HCC) is one of the most malignant tumors with the highest incidence and mortality in the world, causing a serious burden on society. Pyruvate kinase M2 (PKM2) is one of the principal metabolic enzymes involved in glycolysis. Studies have shown that PKM2 is highly expressed in HCC and can be translocated to the nucleus, where it interacts with various transcription factors and proteins such as hypoxia-inducible factor-1α, sterol regulatory element-binding protein 1a, signal transducer and activator of transcription 3, nuclear factor erythroid 2-like 2 and histone H3, exerting non-metabolic enzyme functions to regulate the cell cycle, proliferation, apoptosis, immune escape, migration, and invasion, as well as HCC angiogenesis and tumor microenvironment. This review is focused on the recent progress of PKM2 interacting with various transcription factors and proteins affecting the onset and development of HCC, as well as natural drugs and noncoding RNA impacting diverse biological functions of liver cancer cells by regulating PKM2 non-metabolic enzyme functions, thereby providing valuable directions for the prognosis improvement and molecular targeted therapy of HCC in the future.

## 1. Introduction

One of the important hallmark changes of tumors is the metabolic pathway switching from oxidative phosphorylation to high-level aerobic glycolysis, namely the “Warburg effect.”^[1]^ Pyruvate kinase catalyzes the last irreversible reaction of glycolysis - phosphoenolpyruvate (PEP) is converted to pyruvate, which plays a crucial role in glycolysis during tumorigenesis.^[2]^ Pyruvate kinase has 4 tissue-specific expression subtypes (muscle, M1; liver, L; erythrocyte, R; ubiquitous, M2), pyruvate kinase M2 (PKM2) is one of the more extensively studied subtypes and is known as a tumor-specific subtype. Hepatocellular carcinoma (HCC) is one of the most malignant tumors with the highest incidence and mortality in the world, causing a serious burden on society. Therefore, exploring the molecular mechanism of HCC tumorigenesis is of great significance for the diagnosis and treatment of this disease. Studies have found that PKM2 is highly expressed in HCC and closely correlated to tumor staging and prognosis of HCC, which is suggested that PKM2 may be a pivotal modulator in the occurrence and development of HCC.^[3]^ Apart from its metabolic function in glycolysis to regulate HCC occurrence and development, PKM2 also has a non-metabolic role, whereby it affects liver cancer cell biological functions, and its related pathways are targets for preventing and treating HCC.^[4–6]^ This article will review the recent progress of PKM2 interacting with various transcription factors and proteins affecting the onset and development of HCC, as well as natural drugs and noncoding RNA impacting diverse biological functions of liver cancer cells by regulating PKM2 non-metabolic enzyme functions.

## 2. Basic structure and function of PKM2

**Figure 1.**
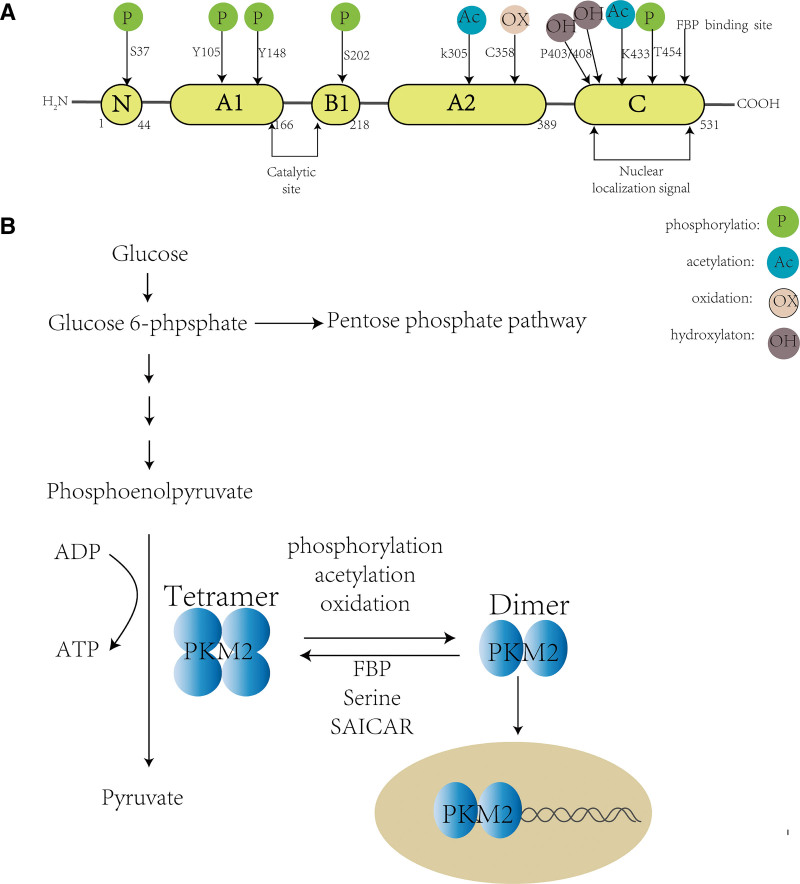
Basic structure and function of PKM2 monomer, dimer, and tetramer. (A) Basic structure of PKM2 monomer (B) Basic function of PKM2 dimer and tetramer. ADP = adenosine diphosphate, ATP = adenosine triphosphate, FBP = fructose 1,6-bisphosphate, PKM2 = pyruvate kinase M2, SAICAR = succinyl-5-aminoimidazole-4-carboxamide-1-ribose 5’-Phosphate.

PKM gene is located on chromosome 15q23 region and can be alternatively spliced into PKM1 or PKM2. PKM2 consists of 531 amino acids, including A, B, and C structural domains, and has multiple sites that can be phosphorylated, acetylated, hydroxylated, and oxidized (see Fig. 1A).^[7,8]^ PKM2 monomer subunits can aggregate to form high metabolic activity tetramers and low metabolic activity dimers. PKM2 tetramers have high affinity with substrate PEP and high pyruvate kinase activity; while low-activity dimer PKM2 acts as a protein kinase or co-transcription factor into the nucleus, promoting tumor development and progression, showing unique non-metabolic effects (see Fig. 1B).^[7]^

The transition between PKM2 dimers and tetramers is regulated by the conformational effects of endogenous and exogenous activators and inhibitors. PKM2 tetramers can be activated by fructose 1, 6-bisphosphate or serine and succinyl-5-aminoimidazole-4-carboxamide-1-ribose 5’-Phosphate conformational effects. While phosphorylation, acetylation, and oxidation of PKM2 Tyr-105, Lys 305, and Cys 358 sites can prevent fructose 1, 6-bisphosphate from binding to tetrameric PKM2, thus keeping PKM2 in dimer form.^[8]^ In addition, interaction with Jumonji C domain-containing protein 5 or p300-mediated PKM2 Lys (K) 433 acetylation can also prevent PKM2 tetramerization, enhancing PKM2 nuclear translocation.^[9]^

PKM2 dimers can act as switches for energy metabolism and material synthesis in the context of glucose metabolism, converting the process of glucose metabolism to PEP pathway, providing carbon sources and redox equivalents for ribose and nonessential amino acid synthesis; in the context of non-glucose metabolism, PKM2 dimers can play different roles at different sites, such as entering the nucleus to regulate gene expression, or attaching to the outer membrane of mitochondria to maintain mitochondrial function, or locating in the endoplasmic reticulum to inhibit endoplasmic reticulum stress.^[10]^

## 3. The effect of the non-metabolic enzyme function of PKM2 in the nucleus on HCC tumorigenesis and development

**Figure 2.**
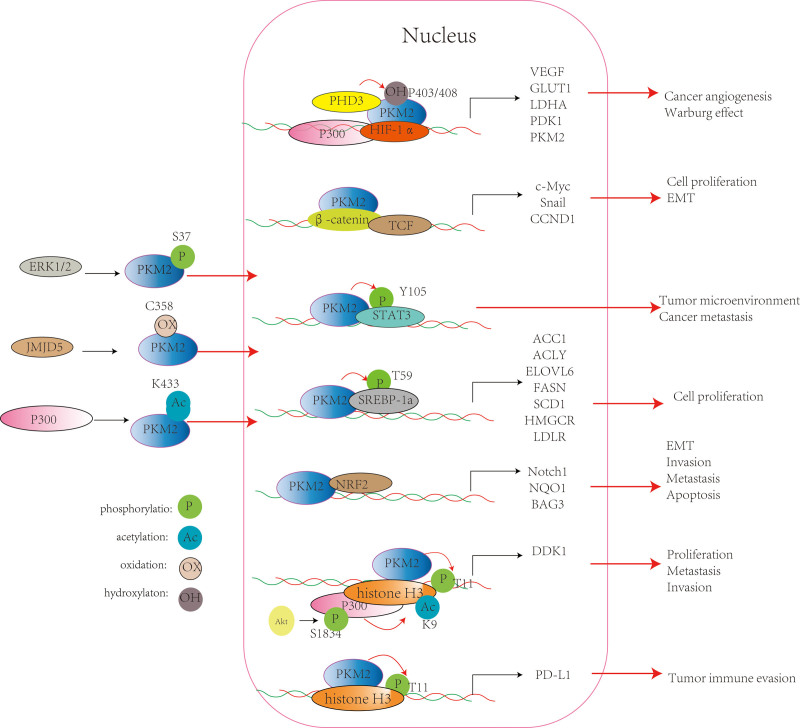
Nuclear PKM2 regulates the expression of related genes affecting HCC tumorigenesis and development. ACC1 = acetyl-CoA-carboxylase 1, ACLY = ATP-citrate lyase, BAG3 = Bcl2-associated athanogene 3, CCND1 = cyclin D1, DKK1 = dickkopf-1, ELOVL6 = elongation of very long chain fatty acids protein 6, ERK1/2 = extracellular regulated protein kinases, FASN = fatty Acid synthase, GLUT1 = glucose transporter-1, HCC = hepatocellular carcinoma, HIF-1α = hypoxia-inducible factor-1α, HMGCR = hydroxymethylglutaryl-CoA reductase, JMJD5 = jumonji C domain-containing protein 5, LDHA = lactate dehydrogenase A, LDLR = low-density lipoprotein receptor, NQO1 = NADPH quinone oxidoreductase 1, NRF2 = nuclear factor erythroid 2-like 2, PDK1 = pyruvate dehydrogenase kinase 1, PD-L1 = programmed cell death-ligand 1, PHD3 = prolyl hydroxylase domain 3, PKM2 = pyruvate kinase M2, SCD1 = stearoyl-CoA desaturase 1, SREBP-1a = sterol regulatory element-binding proteins1a, STAT3 = signal transducer and activator of transcription 3, VEGF = vascular endothelial growth factors.

One of the hotspots of current research is that PKM2 enters the nucleus to exert non-metabolic effects affecting tumor tumorigenesis and development. Hypoxia-inducible factor-1α (HIF-1α) and epidermal growth factor receptor (EGFR) activation can promote PKM2 nuclear translocation.^[9,11]^ Its nuclear translocation depends on its C-terminal nuclear localization signal. When extracellular regulated protein kinases phosphorylate PKM2 S37 site, peptidyl-prolyl cis-trans isomerase NIMA-interacting 1 mediates the isomerization of PKM2 from tetramer to monomer, exposing nuclear localization signal into the nucleus. After entering the nucleus, PKM2 not only acts as a transcriptional co-activator, promoting the activation of transcription factors such as HIF-1α, β-catenin/TCF, signal transducer and activator of transcription 3 (STAT3), sterol regulatory element-binding proteins 1a (SREBP-1a), nuclear factor erythroid 2-like 2 (NRF2), etc, affecting the expression of their downstream target genes; but also acts as a protein kinase phosphorylating histone H3, affecting the transcription of related target genes.^[10]^ Therefore, an instrumental role of nuclear PKM2 in the regulation of gene expression is associated with the development and progression of HCC (see Figure 2).

### 3.1. PKM2/HIF-1α

Tumor cells are in a state of uncontrolled growth, unlimited division, and apoptosis resistance, so they need extra oxygen and nutrient supply. They can adapt to these needs through angiogenesis and metabolic changes. HIF-1α, as a transcription factor, is closely related to the occurrence and development of tumors by regulating the transcription of key genes involved in angiogenesis and Warburg effect.^[12,13]^ Previous studies have focused more on HIF-1α as a transcription factor promoting the transcription of several glycolysis-related genes (glucose transporter-1, lactate dehydrogenase A, pyruvate dehydrogenase kinase 1, and PKM2) to promote HCC glycolysis and thus affect liver cancer cell proliferation.^[14]^ Interestingly, studies have also demonstrated that PKM2 can regulate HIF-1α expression and activation.^[12,15–17]^ Azoitei N et al^[12]^ found that PKM2 entered the nucleus and activated NF-κB/p65 and HIF-1α in hypoxic pancreatic tumors, leading to increased transcription of vascular endothelial growth factors, ultimately triggering vascular endothelial growth factors-A secretion and angiogenesis. Liu WR et al^[15]^ found that knocking down PKM2 in highly metastatic liver cancer cells HCCLM3 could reduce the expression of HIF-1α protein and the phosphorylation of AKT and JUK, while reducing liver cancer cell angiogenesis ability, reducing the number of tubules, number of tubules branching points, and tubule length. These studies suggest that PKM2 may affect HIF-1α expression, and then affect the occurrence and development of tumors including HCC. How does PKM2 affect HIF-1α function? Studies have shown that PKM2 is hydroxylated at proline 403/408 by prolyl hydroxylase domain 3 and hydroxylated PKM2 binds to the HIF-1α subunit, enhances the binding of HIF-1α to p300, and recruits p300 to hypoxia response element to promote transactivation of HIF-1α target genes, thereby also promoting PKM2 transcription.^[16]^ Therefore, PKM2 and HIF-1 form positive feedback. Furthermore, recent research reported that the recruitment of HIF-1α and p300 mediated by nuclear PKM2 under hypoxia in breast cancer cells also caused the upregulation of 6-phosphofructo-2-kinase/fructose-2, 6-bisphosphatase 3 a glycolytic enzyme and positively associated with cancer progression and aggression).^[17]^ These studies suggest that nuclear PKM2 can promote glycolysis by activating HIF-1α, potentially playing a role in various biological functions of HCC. These findings highlight the potential of PKM2/HIF-1α as a target for anti-HCC therapies.

### 3.2. PKM2/β-catenin

β-catenin is a key molecule of Wnt/β-Catenin signaling pathway. After entering the nucleus, it can bind to transcription factor TCF, increase the transcription of target genes c-MYC, snail, and cyclin D1 (CCND1), and promote liver cancer cell proliferation and epithelial-mesenchymal transition (EMT).^[18]^ Yang W et al^[19]^ first found that EGFR could promote PKM2 nuclear entry in glioma cells. Nuclear PKM2 K433 could bind to the Y333 site of β-catenin, which is phosphorylated by c-Src, thus removing histone deacetylase 3 from CCND1 promoter. As a result, the transcription of CCND1 is up-regulated, leading to an increase in the proliferation of glioma cells. Fan FT et al^[18]^ also found that EGFR-activated nuclear PKM2 could increase β-catenin transactivation in liver cancer cells, promoting the transcriptional expression of its target genes and participating in the EMT process of liver cancer cells. Further studies showed that histone deacetylase 8 could bind and deacetylate the K62 residue of PKM2. The deacetylation of K62 promotes the nuclear translocation of PKM2 and binding to β-catenin, thereby promoting CCND1 gene transcription and growth of HCC cells.^[20]^ Therefore, these results suggest that PKM2/β-catenin signaling may become a target for the treatment of HCC. The latest study found that tumor suppressor gene Rho-GTPase-activating protein 24 (ARHGAP24) can inhibit liver cancer cell proliferation, induce liver cancer cell G0/G1 phase arrest, and inhibit liver cancer cell invasion and migration. The underlying mechanism might be related to its inhibition of β-catenin transactivation. Further studies have found that ARHGAP24 binds to PKM2 and recruits a new E3 ubiquitin ligase WWP1, promoting PKM2 degradation, thus inhibiting PKM2-mediated β-catenin transactivation, inhibiting liver cancer cell proliferation, migration, and other biological functions.^[4]^ This suggests that the ARHGAP24/WWP1/PKM2/β-catenin axis can inhibit HCC tumorigenesis and development and can be used as a new approach for treating HCC.

### 3.3. PKM2/STAT3

Nuclear PKM2 can phosphorylate transcription factor STAT3 at Y105 site, reshaping the HCC tumor microenvironment and promoting HCC migration.^[21,22]^ Hou PP et al^[21]^ found that ubiquitin-like modification of PKM2 in liver cancer cells led to PKM2 interaction with arrestin domain-containing protein 1, which promoted the release of extracellular granules containing PKM2. The extracellular granules containing PKM2 not only induced monocyte differentiation into macrophages, but also induced nuclear STAT3 phosphorylation, up-regulated differentiation-related transcription factors, and promoted liver cancer cell proliferation, and migration by changing liver cancer cell immune microenvironment. Yu Z et al^[22]^ found that knocking down PKM2 in liver cancer cells inhibited cell proliferation, autophagy, invasion, and migration, increased apoptosis, and increased JAK/STAT3 pathway activity. Inhibiting STAT3 activity reversed the effect of knocking down PKM2 on cell migration, suggesting that PKM2-induced liver cancer cell migration is mediated by STAT3. The regulation of STAT3 phosphorylation by PKM2 in these studies is inconsistent, which may be related to the different cell types and downstream targets of STAT3, but they all indicate that PKM2 can regulate STAT3 phosphorylation and affect HCC tumorigenesis and development. Li Q et al^[23]^ showed that nuclear PKM2 can promote gefitinib resistance in colorectal cancer by up-regulating STAT3 activation, but whether it has the same effect in HCC remains to be further studied.

### 3.4. PKM2/SREBP-1a

SREBP-1a is highly expressed in various tumor tissues and acts as a transcription factor. It can transactivate all SREBP target genes such as Acetyl-CoA-carboxylase 1, adenosine triphosphate-citrate lyase, elongation of very long chain fatty acids protein 6, fatty acid synthase, stearoyl-CoA desaturase 1, hydroxymethylglutaryl-CoA reductase and low-density lipoprotein receptor, participating in tumor cell lipid metabolism and affecting various biological functions of tumor cells.^[24]^ Zhao X et al^[25]^ found that nuclear PKM2 promoted the proliferation of liver cancer cells, breast cancer cells, colon cancer cells, and lung cancer cells by phosphorylating nuclear SREBP-1a T59, which prevented SREBP-1a from being ubiquitinated and degraded; and then increased the expression of lipid metabolism-related target genes of SREBP-1a. This study suggests that PKM2 acts as a transcriptional co-activator, promoting the transcriptional activation function of SREBP-1a, thereby promoting liver cancer cell proliferation. Interfering with its binding to SREBP-1a may inhibit HCC tumorigenesis and development.

### 3.5. PKM2/NRF2

NRF2 is a transcription factor that can rapidly translocate and accumulate in the nucleus under stress conditions such as oxidative stress or electrophilic stress, and activate target genes such as Notch1, NADPH quinone oxidoreductase 1, and Bcl2-associated athanogene 3 through antioxidant response element, affecting liver cancer cell EMT, invasion and metastasis and anoikis.^[3,26,27]^ Existing studies have shown that dimeric PKM2 can enter the nucleus and promote NRF2 transactivation in various cells such as astrocytes, lung cancer cells A549, and liver cancer cells.^[28]^ But how PKM2 affects NRF2 has not been reported in the literature. These studies suggest that nuclear PKM2/NRF2 interaction may affect HCC tumorigenesis and development.

### 3.6. PKM2/histone H3

The earliest study found that nuclear PKM2 promoted histone H3 Thr11 phosphorylation in glioma cells, eliminating histone deacetylases 3 transcriptional inhibition of CCND1 and MYC genes, thereby increasing their expression and affecting tumor progression.^[29]^ But whether this reaction is also in HCC has not been reported. Dickkopf-1 (DKK1) is a secreted glycoprotein. Studies have shown that DKK1 mRNA and protein expression are higher in rat HCC cancer tissues than in normal liver or adjacent tissues, and overexpression of DKK1 in human liver cancer cell nude mouse xenografts can promote tumor growth and metastasis, suggesting that DKK1 can promote liver cancer cell proliferation and migration,^[6]^ but how DKK1 is overexpressed in liver cancer cells is currently less studied. Niu J et al^[6]^ further found that EGF treatment increased the expressions of DKK1 mRNA and protein in liver cancer cells, and through gene knockout and phosphorylation site mutation experiments, they found that EGF-activated EGFR can induce PKM2 Ser37 residue phosphorylation and p300 Ser1834 residue phosphorylation through extracellular regulated protein kinases signaling pathway and PI3K-AKT signaling pathway respectively. Phosphorylated PKM2 translocated to the nucleus and phosphorylated histone H3 Thr11 site. Phosphorylated p300 acetylated histone H3 Lys9. Histone H3-Thr11 phosphorylation and Lys9 acetylation synergistically promoted DKK1 gene transcription in liver cancer. In addition, this research group also found that EGFR-activated PKM2 phosphorylated H3-Thr11 can recruit immune checkpoint-related molecule programmed cell death 1 ligand 1 (PD-L1) promoter region to enhance PD-L1 transcription, thereby possibly promoting the formation of immune suppressive tumor microenvironment in HCC.^[30]^ These studies indicate that nuclear PKM2 can promote the expression of genes such as DKK1 and PD-L1 by phosphorylating histone H3, thereby promoting liver cancer cell proliferation, migration, and invasion as well as immune escape.

### 3.7. Other transcription factors or signaling pathways affected by nuclear PKM2

In addition, studies have also found that PKM2 can suppress Hippo signaling pathway by inhibiting LAST1 and YAP phosphorylation, thereby promoting liver cancer cell proliferation, invasion, and migration,^[31]^ but how PKM2 affects LAST1 and YAP phosphorylation remains to be further studied. Although there are reports that nuclear PKM2 can promote octamer-binding transcription factor-4 transactivation, which plays an important role in maintaining pancreatic cancer cell stemness,^[32]^ whether PKM2 can also affect octamer-binding transcription factor-4 transactivation in liver cancer cells, affecting liver cancer cell stemness, has not been reported.

## 4. PKM2 regulates mitochondrial function or endoplasmic reticulum stress

Mitochondria maintain cellular energy balance by dynamic changes of fusion and fission in morphology. The dynamic balance of mitochondria in tumor cells is disrupted, promoting tumor cell proliferation.^[33]^ Studies have found that PKM2 can promote mitochondrial fusion and oxidative phosphorylation (OXPHOS) in lung cancer cells and liver cancer cells, while glycolysis is reduced. Further studies have found that this reaction is related to PKM2 binding to mitofusion2 (MFN2), a key regulatory protein of mitochondrial fusion.^[34]^ In addition, mTOR phosphorylates MFN2 to increase PKM2-MFN2 binding, thereby affecting PKM2-MFN2’s modulation of glycolysis, mitochondrial fusion, and OXPHOS.^[34]^ Therefore, the mTOR-MFN2-PKM2 signaling axis regulates tumor cell growth by coupling glycolysis and OXPHOS. Studies have found that resveratrol inhibits proliferation and colony formation, and induces apoptosis of colon cancer cells and cervical cancer cells. Furthermore, inhibition of PKM2 by resveratrol induces endoplasmic reticulum stress in tumor cells, promotes mitochondrial division, and leads to apoptosis in tumor cells. Overexpression of PKM2 can greatly reverse these effects,^[35]^ suggesting that PKM2 can inhibit endoplasmic reticulum stress and mitochondrial fission, and further affect tumor cell proliferation, but whether it has the same effect in liver cancer remains to be studied.

## 5. The effect of regulating PKM2 non-metabolic enzyme function on HCC tumorigenesis and development

The above studies have demonstrated that the entry of dimeric PKM2 into the nucleus can influence the biological functions of HCC cells through multiple pathways. Therefore, regulating the non-metabolic enzyme function of PKM2 will also play a significant role in intervening in HCC tumorigenesis and development. Currently, more studies are on natural products and noncoding RNA regulating PKM2’s non-metabolic enzyme function to affect HCC tumorigenesis and development.^[36,37]^

### 5.1. Natural products regulate PKM2 non-metabolic enzyme function

Many studies have found that shikonin can inhibit PKM2 expression in various tumors and is a classic PKM2 inhibitor.^[38–40]^ Shikonin can inhibit PKM2 glycolytic function in liver cancer cells^[41]^; but also can suppress PKM2 non-metabolic enzyme function to affect liver cancer cell biological function.^[4,42]^ Zhang Huanhuan et al found that shikonin can inhibit liver cancer cell SMMC-7721 proliferation, induce apoptosis, and its effect may be related to inhibiting nuclear PKM2/prolyl hydroxylase domain 3/HIF-1α complex formation.^[42]^ Liu B et al^[4]^ also found that shikonin inhibited high metastatic liver cancer cell HCCLM3 invasion and migration, induced apoptosis, and induced mitochondrial membrane potential decline, further found that its mechanism may be related to PKM2 inhibition, activated Adenosine 5’-monophosphate -activated protein kinase/peroxisome proliferator-activated receptor γ co-activator-1α pathway, increased tumor cell oxidative stress. These studies suggest that shikonin can inhibit liver cancer cell non-glycolytic function by inhibiting PKM2 to exert an anti-liver cancer effect. But there are also studies that shikonin can also activate PKM2 bypass such as PKM2/NRF2/Bcl2-associated athanogene 3 pathway and PKM2/HIF-1α pathway and further reducing tumor cell apoptosis, increasing glycolysis, making liver cancer cells resistant to its anti-liver cancer effect,^[3,43]^ suggesting that shikonin inhibits one of PKM2’s non-metabolic enzyme pathways, may cause other pathways activation, combined with related pathway inhibitors may enhance its anti-liver cancer effect.

PKM2 is one of the targets of proanthocyanidin B2 (PB2) to inhibit HCC.^[44]^ PB2 can inhibit liver cancer cell proliferation and induce apoptosis in vitro, inhibit glycolysis, and inhibit liver cancer growth in vivo. Further studies have found that PKM2 plays an important role in PB2 anti-liver cancer effect and enhances liver cancer cell sensitivity to sorafenib.^[44]^ PB2 can inhibit PKM2 nuclear translocation, reduce HIF-1α expression, reduce PKM2/HIF-1α pathway activity, inhibit HIF-1α transcriptional activity, and further reduce the expression of tumor-related genes regulated by HIF-1α such as glycolysis-related enzymes and glucose transporters, thereby inhibiting liver cancer cell proliferation.^[44]^

### 5.2. Noncoding RNA regulates PKM2 non-metabolic enzyme function

Long noncoding RNA (lncRNA), circular RNA (circRNA) and microRNA targeting PKM2 to affect HCC tumorigenesis and development are one of the current research hotspots.^[36,45]^ noncoding RNA can target PKM2’s metabolic enzyme function to affect liver cancer cell metabolic reprogramming,^[46–48]^ but also target PKM2’s non-metabolic enzyme function to affect HCC tumorigenesis and development.^[49,50]^

Wang C et al^[49]^ found that lncRNA HULC can increase CCND1 expression by miR675-autophagy-PKM2 pathway, further affecting pRB and P21 WAF1/CIP1 expression and increasing liver cancer stem cell proliferation. Wu Y et al^[50]^ showed that lncRNA MNX1-AS1 induced by c-Myc can promote liver cancer cell proliferation in vivo and in vitro. Further studies found that MNX1-AS1 can promote EGFR activation of PKM2 binding to importin α5, promote PKM2 nuclear translocation and increase liver cancer cell Warburg effect. The possible reason is that PKM2 nuclear translocation promotes HIF-1 transcriptional activity and increases the expression of glycolysis-related enzymes involved in MNX1-AS1-induced liver cancer cell glycolysis. Researcher Zhu Qian found that lncRNA HClnc1 binding to PKM2 can promote liver cancer cell proliferation, invasion, and angiogenesis. Its mechanism may be related to HClnc1 reducing PKM2 ubiquitination modification and increasing PKM2 stability. In addition, it is also related to PKM2 promoting STAT3 phosphorylation and increasing the expression of downstream genes related to tumor proliferation, apoptosis, and migration.^[51]^ The above studies suggest that multiple lncRNAs can promote PKM2 expression or nuclear translocation and enhance its non-metabolic enzyme function involved in HCC tumorigenesis and development, providing new research ideas for HCC prevention and treatment.

Pan J et al^[52]^ found that lncRNA NEAT1 can promote PKM2 transcriptional activation by directly binding to transcription factor FOXP3, promoting liver cancer cell proliferation and metastasis. Wang L et al^[53]^ proved that hypoxia-induced lncRNA DACT3-AS1 can up-regulate PKM2 expression through HDAC2/FOXA3 pathway and promote liver cancer cell migration. But these studies did not further clarify whether lncRNA regulates PKM2’s glycolytic enzyme function or non-metabolic enzyme function to affect HCC tumorigenesis and development, which remains to be further studied.

MiR-4417 and miR-372 can also enhance PKM2’s non-metabolic enzyme function involved in HCC occurrence.^[54,55]^ MiR-4417 inhibits tripartite motif containing 35 expression, increases PKM2 phosphorylation at Tyr-105, promotes liver cancer cell proliferation, and inhibits apoptosis.^[54]^ But how PKM2 Tyr-105 phosphorylation affects liver cancer cell proliferation and apoptosis remains to be further studied. Lin Z et al^[55]^ found that miR-372 can inhibit β-catenin degradation, promote β-catenin/TCF4 transcriptional activity, increase PKM2 expression, make PKM2 dimer translocation into the nucleus and acetylate histone H3 lysine at erbB-2 promoter region, thereby increasing erbB-2 expression and promoting liver cancer occurrence. These studies suggest that microRNA, similar to lncRNA, can regulate PKM2’s non-metabolic enzyme function, which is involved in HCC tumorigenesis and development.

There is currently no report of circRNA targeting PKM2’s non-metabolic enzyme function in HCC. Although there are studies that show that circRNA circMAT2B can activate miR-338-3p/PKM2 axis to enhance the glycolysis process under hypoxia and promote liver cell carcinoma growth,^[48]^ whether it can also regulate PKM2’s non-metabolic enzyme function remains to be further studied.

## 6. Conclusion

This article reviews the nuclear PKM2 by directly regulating transcription factor activity as a transcriptional co-activator to regulate gene expression, playing a key role in the epigenetic regulation of gene transcription. Its non-metabolic enzyme function either directly regulates the expression of tumor-related genes, or indirectly promotes the glycolysis process by HIF-1 action, involved in various aspects of HCC occurrence and development, including cell cycle, proliferation, apoptosis, migration and invasion, angiogenesis, immune escape, tumor microenvironment, and so on. Natural products and noncoding RNA regulate PKM2’s non-metabolic enzyme function and affect HCC progression. But what cellular environment can promote PKM2 into liver cancer cell nucleus, what factors can affect PKM2’s oligomeric state (dimer or monomer) in mitochondria and extracellular space, whether oligomer regulation depends on organelle specificity and other issues are not very clear, need further research. We believe that in the near future, with the solution to these problems, PKM2 will possibly become a molecular marker of HCC diagnostic and prognostic, which may be of great importance for the therapeutic targeting of HCC.

## Author contributions

**Conceptualization:** Shuangxia Zhang, Zhangxiu Liao.

**Writing – original draft:** Shuangxia Zhang, Zhangxiu Liao.

**Writing – review & editing:** Shubo Li, Ying Luo.
